# Long-Term Outcomes of Nasopharyngeal Carcinoma in 148 Children and Adolescents

**DOI:** 10.1097/MD.0000000000003445

**Published:** 2016-04-29

**Authors:** Suying Lu, Hui Chang, Xiaofei Sun, Zijun Zhen, Feifei Sun, Jia Zhu, Juan Wang, Junting Huang, Ru Liao, Xiaofang Guo, Lixia Lu, Yuanhong Gao

**Affiliations:** From the State Key Laboratory of Oncology in South China (SL, HC, XS, ZZ, FS, JZ, JW, JH, RL, XG, LL, YG); Collaborative Innovation Center of Cancer Medicine (SL, HC, XS, ZZ, FS, JZ, JW, JH, RL, XG, LL, YG); and Departments of Pediatric Oncology (SL, XS, ZZ, FS, JZ, JW, JH, RL, XG) and Radiation Oncology (HC, LL, YG), Sun Yat-sen University Cancer Center, Guangzhou, Guangdong, China.

## Abstract

The aim of this study was to investigate the survival and long-term morbidities of nasopharyngeal carcinoma (NPC) in children and adolescents.

We retrospectively reviewed children and adolescents with NPC treated at Sun Yat-sen University Cancer Center from February 1991 to October 2010, where the prognostic factors and long-term effects of therapy were analyzed.

A total of 148 patients were identified. The median age was 15 years old (range, 5–18 years) and the male to female ratio was 3.6:1. Most of the tumor histopathology was undifferentiated nonkeratinizing carcinoma (97.3%). The number of patients staged with IVa, IVb, IVc, III, and II were 45 (30.4%), 12 (8.1%), 5 (3.4%), 70 (47.3%), and 16 (10.8%), respectively. For the whole series with a median follow-up of 81 months (range, 6–282 months), the 5-year overall survival (OS) and disease-free survival (DFS) ratios were 79.3% and 69.7%, respectively. We observed significant differences in the 5-year OS (81.1% vs 25.0%, *P* = 0.002) and the DFS rates (72.2% vs 0.0%, *P* = 0.000) between patients with stage II to IVb disease and stage IVc disease. For patients with stage II, III, IVa, and IVb disease, we found a high radiation dose (dose > 66 Gy to the primary lesion) would not significantly improve the survival compared to the sub-high radiation dose group (dose = 60–66 Gy to the primary lesion), even considering the type of radiation therapy technologies. However, the incidences of sequelae (grades I–IV) in patients with high radiation dose were apparently higher than those in patients with low radiation dose.

Considering the late sequelae, a dose of 60 to 66 Gy to the primary lesions seems to be enough for children and adolescents with NPC.

## INTRODUCTION

Nasopharyngeal carcinoma (NPC) is an unusual malignancy in children, which is only 1% to 5% among all pediatric cancers.^1,2^ The annual incidence of NPC is about 0.1 to 1.5 per million in United States, 1.0 per million in North Africa, 2.0 per million in Southeast Asia, and 2.5 per million in Hong Kong.^2^ It is distinguishable from the adult form of the disease due to its different characteristics in epidemiology (close association with Epstein–Barr virus infection and high incidence of locoregionally advanced disease^1^), clinical manifestations (cervical lymphadenopathy is the most common symptom), and pathology (World Health Organization [WHO] type III, undifferentiated carcinoma, is the most common histology type).^1^ Generally, treatment for pediatric patients is extrapolated from the guidelines tailored for adult patients. That is because children and adolescents are usually excluded from adult clinical trials because of the strict age cutoffs.^3^ The treatment strategy for adults mainly consists of high-dose radiotherapy. Undifferentiated NPC is very sensitive to radiation. Combined with radiotherapy and chemotherapy, the 5-year survival has been reported as 55% to 90% in most pediatric series. However, systemic diseases and the late sequelae of radiation cannot be ignored, since endocrine dyscrasia, hearing disorder, bone demineralization, growth retardation, dental problems, life-long dry mouth, and secondary malignancy have been often reported,^2,4–9^ and these sequelae are more pronounced in younger patients.^5–7^ What is more, most of these long-term treatment-related morbidities have been mainly suggested to be related with radiotherapy. Because the most published series are small and the long-term outcomes of children with NPC have not been well characterized. Here we performed a retrospective review of all children and adolescent with NPC treated at our institution during the past 19 years to investigate the long-term survival and morbidities in an endemic area, as well as the factors associated with clinical outcomes.

## MATERIALS AND METHODS

### Patients

The patients were pathologically diagnosed NPC and treated in Sun Yat-sen University Cancer Center from February 1991 to October 2010. All patients aged no more than 18 years were enrolled in this study. All patients had detailed medical records, regarding their clinical history and examinations. Specific printed forms included clinical manifestations, histopathology, diagnostic work-up, therapy, and follow-up were used for data collection from each patient's records. We restaged all patients according to the radiology records (magnetic resonance imaging [MRI] of head and neck, whole-body bone scan, and thoracoabdominal computed tomography scan or chest radiograph plus abdominal ultrasonography) and the Union for International Cancer Control (UICC)/American Joint Committee on Cancer TNM classification version 2009 of NPC. This study was approved by the ethics committee of our hospital. All the patients or their legal guardians signed the informed consent before treatment. As this is a retrospective study, the ethical approval was not necessary.

### Treatment

In this study, treatment strategy of patients was based on National Comprehensive Cancer Network Guidelines. Early-stage (stage I and II) disease was treated with radiotherapy alone. Advanced-stage disease (stage III and IV) was treated with combination of radiotherapy and chemotherapy. The radiotherapy technology consisted of conventional radiotherapy (CRT) and intensity-modulated radiotherapy (IMRT). Most of the patients had received CRT between 1991 and 2004, and IMRT has become the standard strategy for NPC patients in our hospital since 2004.

### Evaluation Criterion

Treatment response was evaluated based on the WHO Criteria in Solid Tumors. A complete remission was defined as no evidence of disease, a partial remission was defined as a decrease of more than 50% in disease, a stable disease was defined as <50% response of the tumor, and more than 25% increment of the tumor size or appearance of new lesions was defined as progressive disease. Chemotherapy toxicity was evaluated based on National Cancer Institute Common Terminology Criteria for Adverse Events (NCI-CTCAE) version 3.0. Radioactive damage on organs was modified from the Toxicity Criteria of the Radiation Therapy Oncology Group (RTOG).

### Endpoints

Acute and late toxicities, disease-free survival (DFS), overall survival (OS), loco-regional control survival (LRCS), and metastasis-free survival (MFS) were the endpoints reviewed in this study. The primary endpoint was DFS, which was defined as the time from study entry to the first progression at any site, recurrence, second malignancy, death, or last disease free visit (months). For OS, the time from study entry to death or until the last follow-up was calculated. LRCS time was measured and calculated from the 1st day of study entry to the first loco-regional failure, while MFS was defined as the time interval from study entry to distant metastasis or until the last reported contact.

### Follow-Up

All patients received a standard follow-up program in our hospital every 3 months during the 1st year, every 6 months in the 2nd year, every year thereafter. History-taking recording and complete head and neck examination were performed in the outpatient department. In this program, fiberscopy was performed at every visit. And nasopharyngeal and cervical MRI was performed 6 months after treatment and at every annual checkup. The data about symptoms and occurrence date of complications after treatment were obtained from the patients’ medical records.

### Statistical Analysis

OS, DFS, LRCS, and MFS were calculated using Kaplan–Meier analysis, in which survival differences were compared with the log-rank test. Prognostic factors were analyzed through the Cox proportional hazards regression model. Chi-squared tests were used to compare the incidences of treatment comorbidities between different groups of patients, which were divided by radiation dose to primary lesion. A difference with 2-sided *P* value of <0.05 was considered to be statistically significant. All calculations were performed with SPSS statistics version 16.0 (SPSS Inc., Chicago, IL).

## RESULTS

### Clinical Characteristics

Between February 1991 and October 2010, 150 patients were enrolled in this study. Two patients before February 2010 who had no complete treatments were excluded from this study. Thus, the number of patients for analysis was 148. Patients’ baseline clinical characteristics were shown in Table 1. The median age of the patients was 15 years (range, 5–18 years) and the male:female ratio was 3.6:1. Most of the tumor histopathology was undifferentiated nonkeratinizing carcinoma (144 patients, 97.3%). The cancer staging distribution of these patients was 10.8% in stage II, 47.3% in stage III, 30.4% in stage IVa, 8.1% in stage IVb, and 3.4% in stage IVc, respectively. There was no patient with stage I disease.

**TABLE 1:**
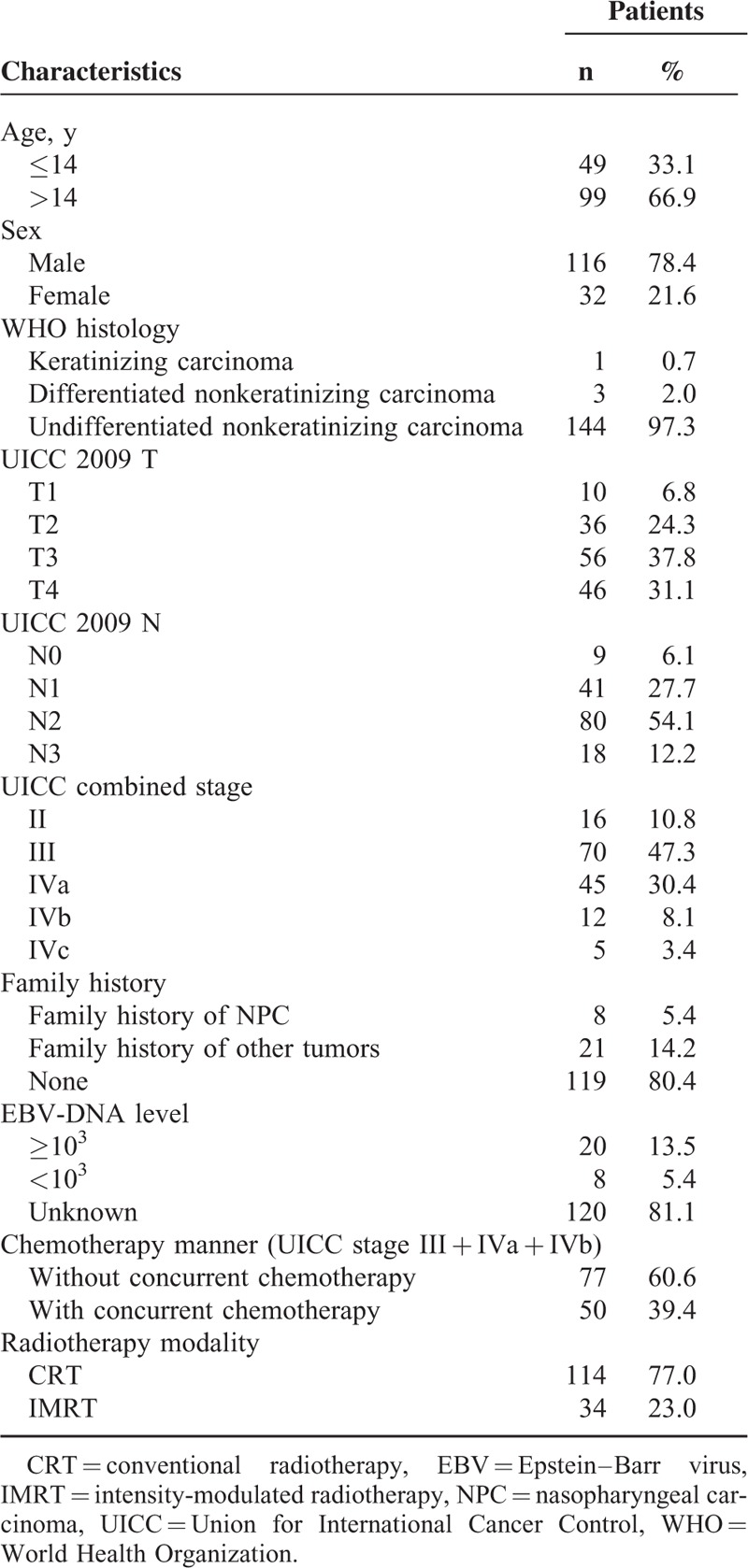
Clinical Characteristics of the 148 Children and Adolescents With Nasopharyngeal Carcinoma

### Treatment

CRT had been used in 114 patients, and IMRT in 34 patients. The median radiation dose was 70 Gy (range, 60–84 Gy) to the primary tumor, and 60 Gy (range, 46–73 Gy) to the cervical lymph nodes. Except for patients with stage IVc disease, 38 patients received a dose of 60 to 66 Gy to the primary lesion, and 104 patients received >66 Gy. All patients were treated with conventional fractionation of 2 Gy per fraction at a dose of 5 daily fractions per week for 7 weeks in total.

Chemotherapy was added to radiation for 118 patients (79.7%). Chemotherapy methods consisted of neoadjuvant chemotherapy (N = 42); concurrent chemotherapy (N = 13); adjuvant chemotherapy (N = 10); neoadjuvant and concurrent chemotherapy (N = 29); concurrent and adjuvant chemotherapy (N = 5); neoadjuvant and adjuvant chemotherapy (N = 12); and neoadjuvant, concurrent, and adjuvant chemotherapy (N = 7). Except for patients with stage IVc disease, 52 patients received concurrent chemotherapy and 91 patients did not received concurrent chemotherapy.

Regimens of neoadjuvant chemotherapy included PF (cisplatin + fluorouracil, N = 68), PBF (cisplatin + bleomycin + fluorouracil, N = 7), TP (taxol + cisplatin, N = 5), and others (N = 9). Regimens of concurrent chemotherapy included cisplatin (N = 34), PF (N = 16), and others (N = 2). Regimens of adjuvant chemotherapy included PF (N = 16), tegadifur (N = 4), and others (N = 14).

### Survival

**FIGURE 1:**
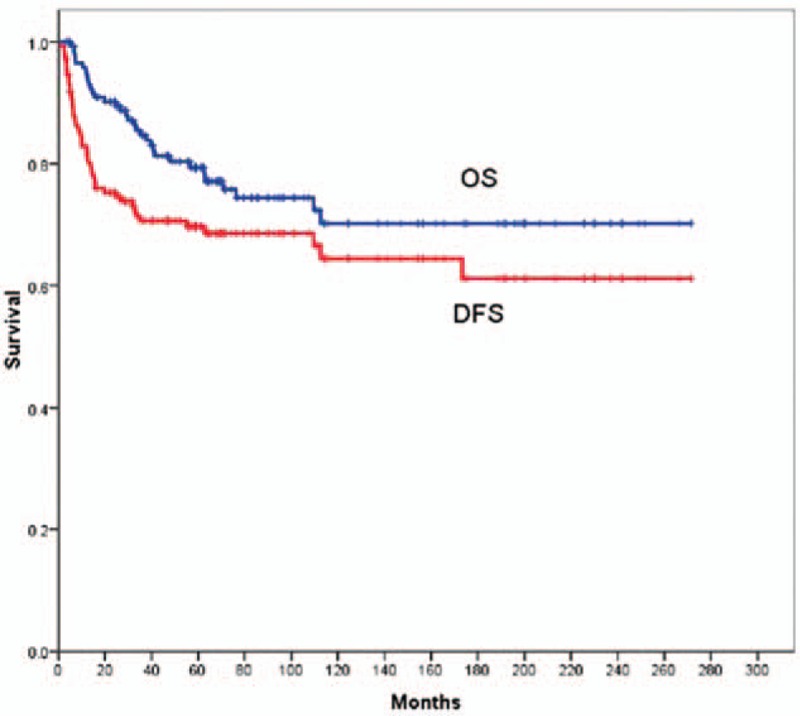
Kaplan–Meier estimates of OS and DFS are shown for all the 148 children and adolescents with nasopharyngeal carcinoma. DFS = disease-free survival, OS = overall survival.

**FIGURE 2:**
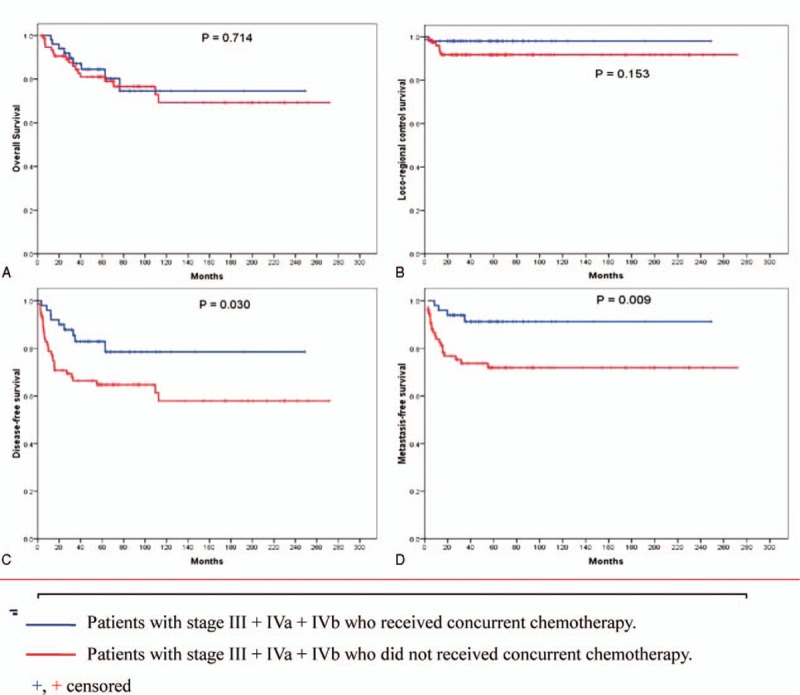
Kaplan–Meier estimates of OS (A), LRCS (B), DFS (C), and MFS (D) are shown for patients with stage III + IVa + IVb who received concurrent chemotherapy or not. DFS = disease-free survival, LRCS = loco-regional control survival, MFS = metastasis-free survival, OS = overall survival.

The last follow-up date was June 10, 2014. After a median follow-up of 81 months (range, 6–282 months), 115 patients (77.7%) were alive and without disease. For the whole series, the 5-year OS and DFS were 79.3% and 69.7%, respectively (Figure 1). Importantly, significant differences were found on the 5-year OS between patients with stage II to IVb disease and stage IVc disease (81.1% vs 25.0%, *P* = 0.002), and on the 5-year DFS (72.2% vs 0.0%, *P* = 0.000). Here, the 5-year OS for patients with stage II, III, and IVa to IVb diseases were 72.2%, 79.3%, and 86.3%, respectively (*P* = 0.904), and the 5-year DFS was 74.0%, 71.2%, and 73.0%, respectively (*P* = 0.972). The results of log-rank test were shown in Table 2. There were no considerable differences between patients with stage III and stage IV received radiotherapy combined with chemotherapy and radiotherapy alone on the 5-year OS (84.4% vs 79.7%, *P* = 0.933), or on the 5-year DFS (70.0% vs 68.7%, *P* = 0.854). Except for patients with stage II and stage IVc disease, 50 patients received the concurrent chemotherapy, while other 77 patients did not. Nevertheless, we did not observe any visible difference between the patients with and without the concurrent chemotherapy regarding the 5-year OS or LRCS. However, a large impact on the 5-years DFS and MFS by the concurrent chemotherapy was observed (Figure 2).

**TABLE 2:**
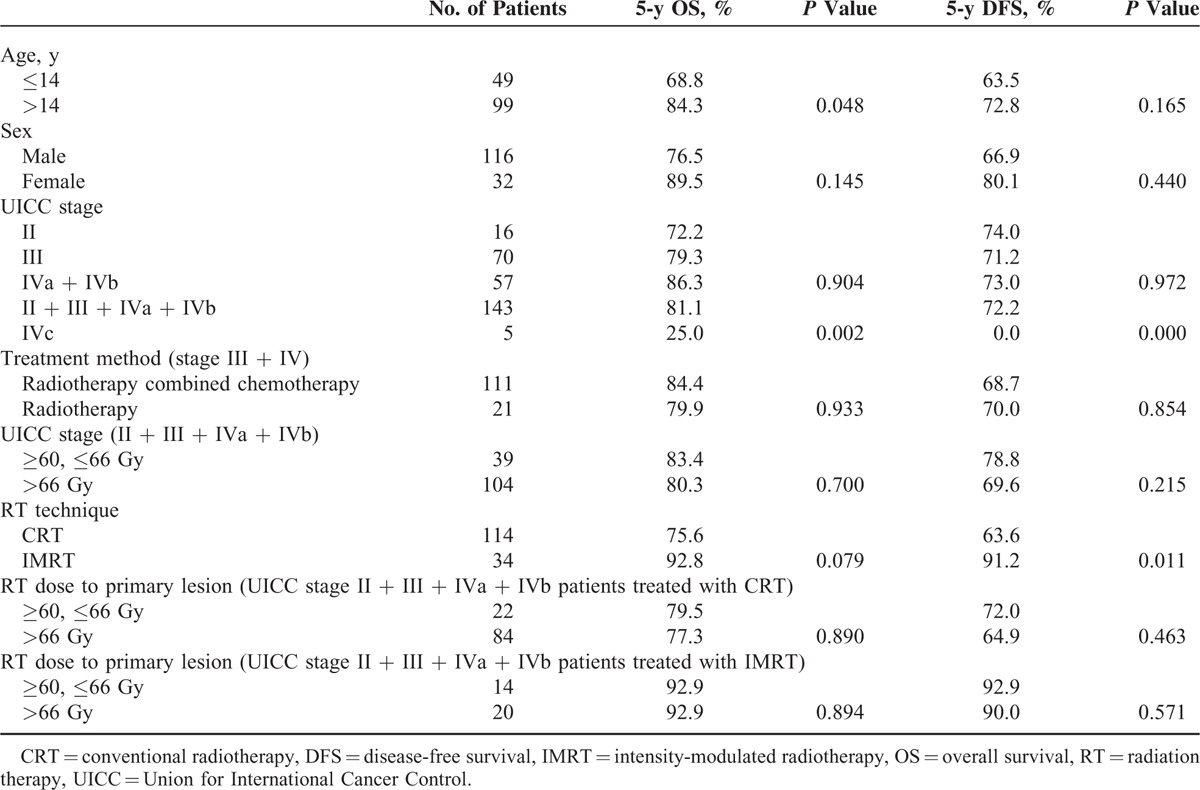
Five-Year OS and DFS for 148 Children and Adolescents With Nasopharyngeal Carcinoma

For patients with stage II, III, IVa, and IVb, high radiation (dose > 66 Gy to the primary lesion) or the sub-high radiation (dose = 60–66 Gy to the primary lesion) did not cause significant difference in survival. The 5-year OS was 80.3% and 83.4% (*P* = 0.700), and the 5-year DFS was 69.6% and 78.8% (*P* = 0.215) for the high and sub-high radiation dose groups, respectively. Taking the radiation therapy into account would not cause any difference between these 2 groups: for the patients treated with CRT, the 5-year OS was 77.3% and 79.5%, respectively (*P* = 0.890), and the 5-year DFS was 64.9% and 72.0%, respectively (*P* = 0.463); for the patients treated with IMRT, the 5-year OS was 92.9% and 92.9%, respectively (*P* = 0.894), the 5-year DFS was 90.0% and 92.9%, respectively (*P* = 0.571).

In multivariate analysis, patients older than 14 years old and stage IVc disease were the independent adverse prognostic factors for OS (the *P* value, 0.032 and 0.012) and DFS (the *P* value, 0.017 and 0.000). Considering the radiotherapy, IMRT was the independent favorable prognostic factor for DFS (the *P* value, 0.025) but not for OS (the *P* value, 0.104).

### Relapse and Metastasis

Among all the 148 patients, we found 10 patients had relapsed, including 4 local recurrences and 6 regional recurrences. And a total of 32 patients had metastases, including 14 bone metastases, 8 lung and mediastinum metastases, 6 lung metastases, and 2 liver metastases. However, no patients relapsed among the 34 patients treated with IMRT. Moreover, metastasis was also largely reduced by IMRT, distant metastasis had only occurred in 4 patients.

### Toxicity

In this study, main acute toxicities were grade I/II hematologic toxicity, and grade I/II mucositis. The most common sequelae were xerostomia, neck fibrosis, tinnitus or hearing loss, trismus, glossolalia, radiation encephalopathy, anxiety, luteinizing hormone/follicle stimulating hormone (LH/FSH) deficiency, hypothyroidism, pulmonary fibrosis (mainly in the apex), growth hormone (GH) deficiency, and secondary malignancy. We found that the incidences of sequelae (grade I–IV) in patients with high radiation dose were apparently higher than those in patients with low radiation dose (Table 3).

**TABLE 3:**
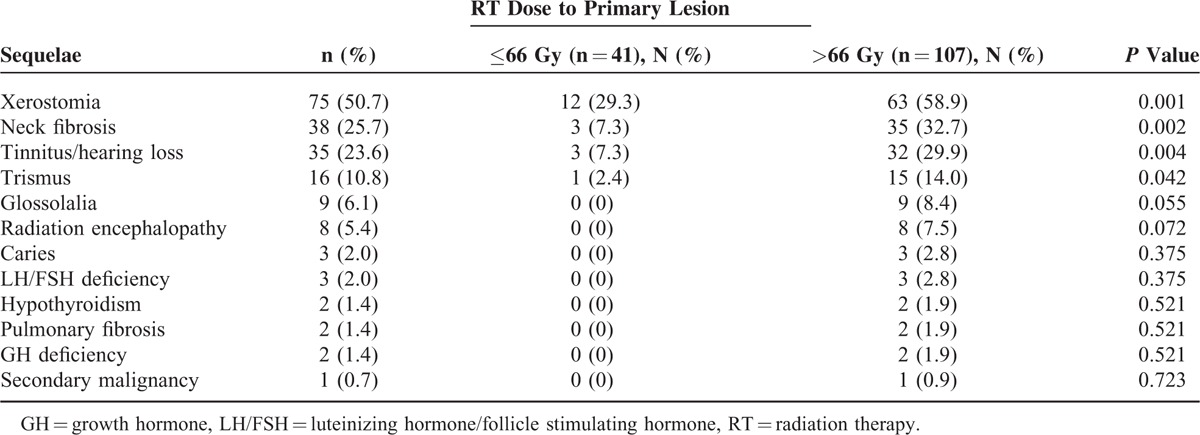
Long-Term Toxicities of All the 148 Children and Adolescents With Nasopharyngeal Carcinoma

## DISCUSSION

Though NPC is a rare disease, South China is an endemic area. NPC in childhood was 2.5% among all the NPC cases and 1.6% of all pediatric solid tumors treated in our department during the period we examined. The NPC incidence for males was apparently higher than that for females, with a ratio of 2.5:1 in young patients^7^ and 2 to 3:1 in the general population.^8^ In this study, we recorded a male to female ratio of 3.6:1, similar to these studies.

Age is one of the factors that affect prognosis. A study from Pakistan reported that the outcome was better in younger children. There was a significant difference in the OS (*P* = 0.001) and the event-free survival (EFS) (*P* = 0.057) in patients diagnosed with NPC under 14 years old and those between 14 and 18 years.^10^ Another 2 studies showed a similar impact of age on OS and EFS.^2,11^ By dividing the patients into a younger children group (under 14 years old) and an older children group (between 14 and 18 years old), our results showed an apparent difference in the 5-year OS between the younger children and the older children (68.8% vs 84.3%, *P* = 0.048), in line with literatures.^2,10,11^ Moreover, patients under 14 years old appeared to have a better OS and DFS according to the multivariate analysis. In addition, the incidence of long-term toxicities for the younger children (51.0%) was significantly higher than those for the older children (33.3%), respectively (*P* = 0.038). Therefore, patients younger than 14 years demonstrated a relative poor outcome at diagnosis than those between 14 and 18 years.

The clinical stage was also reported to have a significant impact on OS (*P* = 0.007) and DFS (*P* = 0.012).^12^ Based on the univariate analysis, Liu et al^13^ reported that the 5-year OS of NPC at stage IV and II to III in childhood and adolescence were 77.1% and 90% (*P* = 0.04), respectively. Regarding the multivariate analysis, stage IVc (*P* = 0.04) was the independent adverse prognostic factors for OS. And through multivariate analysis we found that stage was an independent risk factor for 5-year OS (*P* = 0.012) and 5-year DFS (*P* = 0.000).

To date, the standard therapy for NPC in children generally follows the guidelines established for adults. No standard total radiation dose applied to the tumor has been established, especially combined with the chemotherapy. Ozyar et al^11^ reported that a dose larger than 66 Gy had a better locoregional relapse-free survival (*P* = 0.01) in multivariate analysis. Other studies also showed that a dose of 64 to 80 Gy or an even higher dose was necessary.^14,15^ However, literatures also reported that higher radiation dose (>70 Gy) did not promise a better local control or survival.^12,16^ It could be even worse that higher radiation doses in children might cause a long-term morbidity.^17^ Indeed, there are reports showing that higher radiation dose would inevitably damage normal tissue, causing a higher incidence of severe late sequelae and second malignancies.^1,4^ The correlation between the radiation dose and the survival time is not clear yet. In our study, the difference in survival between the high radiation dose group (dose > 66 Gy to the primary lesion) and the low radiation dose group (dose = 60–66 Gy to the primary lesions) for patients with stage II, III, IVa, and IVb was less pronounced. The 5-year OS was 87.0% and 80.3% (*P* = 0.487), and the 5-year DFS was 84.1% and 70.6% (*P* = 0.121) for the high and low radiation dose groups, respectively. However, the incidences of sequelae in the high radiation dose group were significantly higher than those in the low radiation dose group. Therefore, a dose of 60 to 66 Gy to the primary lesions may be enough for children and adolescents with NPC.

NPC is a highly radiosensitive and chemosensitive tumor. Radiation therapy remains as the mainstay of treatment for adult patients with early stage now.^18–20^ For those with advanced disease, addition of chemotherapy has been demonstrated to be beneficial.^2,21,22^ With radiotherapy alone, the 5-year OS rate is about 20% to 60% in most pediatric series.^4^ Recently, most pediatric patients have received a combination of radiotherapy and chemotherapy, which results in the 5-year OS rates varying from 55% to 90%, and the DFS rates varying from 60.6% to 77.0%.^14,16,23–30^ In this study, the 5-year OS and DFS rates of the whole series were 79.3% and 72.9%, respectively. However, it is surprising that we did not observe any difference in the 5-year OS and 5-year LRCS rates for patients with stage III, IVa, and IVb diseases, whether they had received concurrent chemotherapy or not, but a large impact on the 5-year DFS and MFS rates was observed. So, it is certain that concurrent chemotherapy plays an important role in the treatment for patients with local advanced disease.

Although the 5-year OS rates of NPC have increased with combined therapy modalities, late complications could be a major concern. Cheuk et al^9^ found a 15-year cumulative incidence of any morbidity at 84% (53% for hearing loss, 43% for hypothyroidism, and 14% for GH deficiency), associated with the radiation dose. Sumitsawan et al^31^ reported that the most common complication by radiation was dryness of mouth (97.5%), followed by hearing impairment (82.5%). A study from China reported that the late damages affecting life qualities were found in 26% of irradiated children, particularly among those under 15 years old^9^; 28% of the children who were irradiated had serious long-term treatment-related morbidities.^2^ Significant dryness of the mouth was the most frequent early complaint, which occurred in 95% of the patients. Another study showed that the main late effects included subcutaneous fibrosis (54%), xerostomia (41%), and sensorineural hearing loss (38%).^2^ In this study, the most common late morbidities were xerostomia (50.7%), neck fibrosis (25.7%), and tinnitus/hearing loss (23.6%). Late complications were more frequent in patients younger than 15 years, particularly in those younger than 12 years (68% vs 54%).^2^ Another study also showed that young patients were at a higher risk of developing therapy-related complications, including second cancer.^32^ Compared with CRT, IMRT may be a good way to reduce these toxicities. IMRT has been reported to produce excellent treatment results and carry a decreased risk of long-term side effects in numerous literatures.^33^ Laskar et al^7^ reported that the median time from starting radiotherapy to the development of grade II toxicity was considerably delayed in children treated with IMRT. A significant reduction in acute grade III toxicities of the skin, mucous membrane, and pharynx was reported with the use of IMRT. IMRT seems to be an effective modality for the treatment of pediatric NPC with a significant reduction in toxicity without compromising disease control.

Several limitations should be addressed for our series. First, this is a retrospective study. Second, this study is based on a single center data, our results should be further validated by additional data sets. The third is a relatively short follow-up time, some of the late toxicities had not been observed. For instance, cranial nerve palsy often occurs nearly after 8 years in average, as reported by Kong et al.^34^ In addition, the risk of GH deficiency, FH/FSH deficiency, and hypothyroidism increased with time and might be diagnosed after 30 years.^10^

## CONCLUSIONS

In conclusion, the therapeutic outcomes of radiotherapy combined with chemotherapy on children and adolescents with NPC in this study were satisfactory. Due to the high incidences of the late sequelae by radiation, a dose of 60 to 66 Gy to the primary lesions is suggested for children and adolescents with NPC according our study. In addition, it seems to be important to choose an appropriate treatment. Compared with CRT, IMRT appears to be an effective modality for the treatment of pediatric NPC with a significant reduction in toxicity without compromising disease control.
